# CRDB: Database of Chemosensory Receptor Gene Families in Vertebrate

**DOI:** 10.1371/journal.pone.0031540

**Published:** 2012-02-29

**Authors:** Dong Dong, Ke Jin, Xiaoli Wu, Yang Zhong

**Affiliations:** 1 Institute of Molecular Ecology and Evolution, iAIR, East China Normal University, Shanghai, China; 2 School of Life Sciences, Fudan University, Shanghai, China; 3 Donnelly Centre for Cellular and Biomolecular Research, University of Toronto, Toronto, Ontario, Canada; 4 Institute of Biodiversity Science and Geobiology, Tibet University, Lhasa, China; Laboratoire Arago, France

## Abstract

Chemosensory receptors (CR) are crucial for animals to sense the environmental changes and survive on earth. The emergence of whole-genome sequences provides us an opportunity to identify the entire CR gene repertoires. To completely gain more insight into the evolution of CR genes in vertebrates, we identified the nearly all CR genes in 25 vertebrates using homology-based approaches. Among these CR gene repertoires, nearly half of them were identified for the first time in those previously uncharacterized species, such as the guinea pig, giant panda and elephant, etc. Consistent with previous findings, we found that the numbers of CR genes vary extensively among different species, suggesting an extreme form of ‘birth-and-death’ evolution. For the purpose of facilitating CR gene analysis, we constructed a database with the goals to provide a resource for CR genes annotation and a web tool for exploring their evolutionary patterns. Besides a search engine for the gene extraction from a specific chromosome region, an easy-to-use phylogenetic analysis tool was also provided to facilitate online phylogeny study of CR genes. Our work can provide a rigorous platform for further study on the evolution of CR genes in vertebrates.

## Introduction

In vertebrate, chemosensory receptors (CR) can detect different odorant and taste molecules in the environment and play important roles for the survival of animals because it can help them to find food, identify mates and avoid danger, etc. [1], [2], [3], [4]. Vertebrate CRs are all G protein-coupled receptors (GPCRs) characterized by their seven trans-membrane regions, and CR genes are diversely encoded in animal genomes. CRs mainly contain six different multigene families in vertebrate animals: olfactory receptor (OR), vomeronasal receptor type 1 and type 2 (V1R and V2R), trace amine-associated receptor (TAAR) and taste receptor type 1 and type 2 (T1R and T2R). OR, V1R, V2R and TAAR genes encode pheromone receptors, among which OR and TAAR genes are mainly expressed in the main olfactory epithelium (MOE), and V1R and V2R are encoded in the vomeronasal organ (VNO). The taste receptors, T1R and T2R, are mainly expressed in the taste buds of the tongue [2].

Previous studies have identified many CR genes using PCR-based approaches [5]–[12]. However, these methods have many limitations which cannot identify the entire set of CR genes and might overestimate the proportion of potentially functional genes. With the emergence of whole-genome sequences, it is possible for us to identify the nearly complete CR gene repertoires using data-mining methods [3], [4], [13]–[27]. Up to date, myriads of works have identified many CR gene repertoires and reported that the numbers of CR genes vary extensively among different species [2]. For examples, rats have nearly ∼1,800 OR genes, whereas humans only have ∼800 OR genes [20]; Herbivorous (such as cows and horses) and omnivorous animals (such as humans and mice) have larger T2R gene repertoires than that of carnivores (such as dogs) [14], [23]; Zebrafishes have more than 100 TAAR genes, whereas humans have not more than 10 TAAR genes [26]. Moreover, the faction of pseudogenes also varies extensively among different species [2]. These findings suggested that CR genes are subject to an extreme form of birth-and-death evolution, and are closely related to the survival environments and dietary habits of different species [14], [20]–[23], [25]–[27].

These CR genes provide us an opportunity to better understand their evolution among vertebrate animals. In this work, we identified and annotated nearly the entire sets of CR genes in 25 vertebrate species whose high-coverage genome sequences are available. Furthermore, we constructed a database of CR genes in vertebrates (CRDB, http://zldev.ccbr.utoronto.ca/CRDB/) with the purpose of facilitating CR gene analysis. The product of this effort might become an important repository for CR genes in vertebrates.

## Results and Discussion

### Identification of CR genes in vertebrates

**Figure 1:**
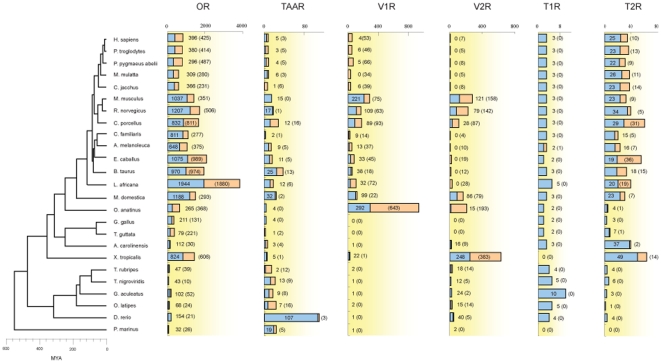
Number of chemosensory receptor genes in vertebrates. The numbers of each bar represent the number of functional (intact) and pseudogenes of OR, TAAR, V1R, V2R, T1R and T2R genes. The blue and orange bars represent the number of functional genes and pseudogenes, respectively.

Using previously published CR proteins as query sequences, we identified the CR genes from 25 vertebrate genome sequences, which can be classified into six different multigene families (see **Materials and methods**). **Figure 1** summarizes the numbers of identified CR intact genes and pseudogenes in each species. It contains a total of 28,596 CR genes, including 23,217 OR genes, 449 TAAR genes, 2,288 V1R genes, 1,902 V2R genes, 80 T1R genes and 660 T2R genes, respectively. The number of CR genes are quite different among these species, which reinforces the concept of an extreme form of ‘birth-and-death’ evolution of CR genes, especially in OR gene family [2]. Many works have reported the extreme form of ‘birth-and-death’ evolution in olfactory and bitter taste receptor genes [2], [13], [14], [20]. To further detect this evolution pattern of CR genes, we performed a further analysis using simulation method. Briefly, we randomly selected 50 genes from 10 species, and then built the phylogenetic tree to detect whether these genes underwent ‘birth-and-death’ evolution. This work performed 1,000 times, and then we found significant ‘birth-and-death’ mechanisms at each time (100% bootstrap value support).

#### (i) OR genes

OR genes form one of the largest multigene families in vertebrate animals. As shown in **Figure 1**, OR genes are present in all vertebrate species, among which 13,396 intact OR genes and 9,821 pseudogenes were identified. The number of OR genes is not equally distributed in each species. It is obviously that mammals contain larger OR gene repertories (400–3,800 genes) than that of fishes and birds (30–100 genes). For example, mice and dogs are considered as macrosmatic animals that rely on the sense of smell, and they contain 1,388 and 1,088 OR genes [20], respectively. In contrast, fishes have only ∼100 OR genes which are much smaller than that of tetrapod animals. Consistent with the concept that birds often have poor sense of smell, limited numbers of OR genes were found in the chicken and zebra finch (342 and 300 genes, respectively). In addition to the previously identified OR genes, we also detected OR genes from some previously uncharacterized species. We found 1,643 OR genes from the guinea pig genome, which is nearly similar to the numbers of OR genes in the mouse and rat. Surprisingly, nearly half of OR genes in the guinea pig are pseudogenes (49% pseudogenes), which are significantly higher than those of the mouse (25%) and rat (29%) [20]. Cows and horses are domesticated herbivores, and they both contain large OR gene repertoires (1,944 and 2,064 genes) and high fractions of pseudogenes (50% and 48%). It was documented that horses and cows are all have strong sense of smell [28], [29], [30]. It raised an interesting question why these animals have large numbers of OR genes and nearly the same number of intact gene and pseudogenes. As pseudogenes can be regarded as relics under the relaxed natural selection, it is possible that some of functional genes have become nonfunctional during the domestication. The giant panda, as a ‘vegetarian carnivore’, contains 1,023 OR genes (648 intact genes and 375 pseudogenes), and indicates that the giant panda still has a strong sense of smell which is concerted with previous findings [31]. Notably, we found that the elephant contains the largest OR gene repertoires (3,824 genes) among vertebrate species, indicating its keener sense of smell than other species.

#### (ii) TAAR genes

TAAR can respond to trace amines. In contrast to OR genes, the number of TAAR genes is smaller (**Figure 1**). For examples, primates have no more than 10 TAAR genes; in the chicken and zebra finch, only three and four TAAR genes were identified. Interestingly, the number of TAAR genes highly diverse in fishes, and an expansion of TAAR genes were found in the zebrafish [26]. The real reason is still unclear, and it is possible that biogenic amines are more important odorants in fishes than that of tetrapod animals.

#### (iii) VR genes

The vomeronasal organ can detect pheromones and is responsible for various behavioral and neuroendocrine responses [25]. Two multigene families of vomeronasal pheromone receptors, V1R and V2R, differ extensively in expression locations and gene structures. Many VR genes have been identified and it has been suggested that V1Rs detect airborne chemicals, whereas V2Rs detect water-soluble molecules [25]. For example, teleost fishes lack V1R genes, but have expanded V2R gene repertoires (20–50 genes) [25]. In this work, we found the guinea pig also has relatively expanded VR gene families (182 V1Rs and 115 V2Rs), which is consistent with the finding that rodents have strong vomeronasal pheromone perceptions. The horse, cow and elephant have small sizes of V1R families (78, 56 and 104 genes respectively), whereas V2R genes totally degenerated in these species. It has been documented that the lizard vomeronasal organ is very poorly supplied with secretion [32], and we only found one V1R genes and 25 V2R genes in the lizard. We did not find any VR genes in the chicken and zebra finch genomes indicating the lack of the vomeronasal system in birds.

#### (iv) T1R genes

Taste perception is essential to diet selection in animals. In vertebrates, T1R and T2R are characterized as sweet/umami and bitter taste receptors, respectively. T1R family contains three members: T1R1 and T1R3 are receptors for sensing umami taste, and T1R2 is receptors for sensing sweet taste [23]. Most mammals contain three T1R genes, except in the horse, platypus and elephant. For examples, the elephant contains five T1R genes, including three T1R2 genes; T1R2 gene is not present in the horse and T1R3 is lost in the platypus. Interestingly, T1R1 gene in the panda has become a pseudogene, which can partly explain why the panda is a ‘vegetarian carnivore’ [33]. Furthermore, T1R2 gene was not found in avian genomes. In teleost fishes, many T1R2 genes were found, indicating the importance of T1R2 genes in teleost fishes.

#### (v) T2R genes

It was reported that many poisonous substances tend to be bitter taste, and the sense of bitter taste can prevent animals from ingesting harmful food [34], [35]. Glendinning and co-workers have reported that plants contain more bitter constituents [35]. Therefore, herbivorous and omnivorous mammals would be expected to need a greater level of bitter taste rejection compared with carnivores. We found that the guinea pig, horse and cow have larger T2R gene repertoires (60, 54 and 33 genes, respectively), whereas the panda and dog have relative smaller T2R gene repertoires (23 and 20 genes, respectively). Moreover, the lizard and frog have large number of lineage-specific T2R genes. By contrast, T2R genes birds and fishes have no more than 10 T2R genes.

### Database of CR genes

**Figure 2:**
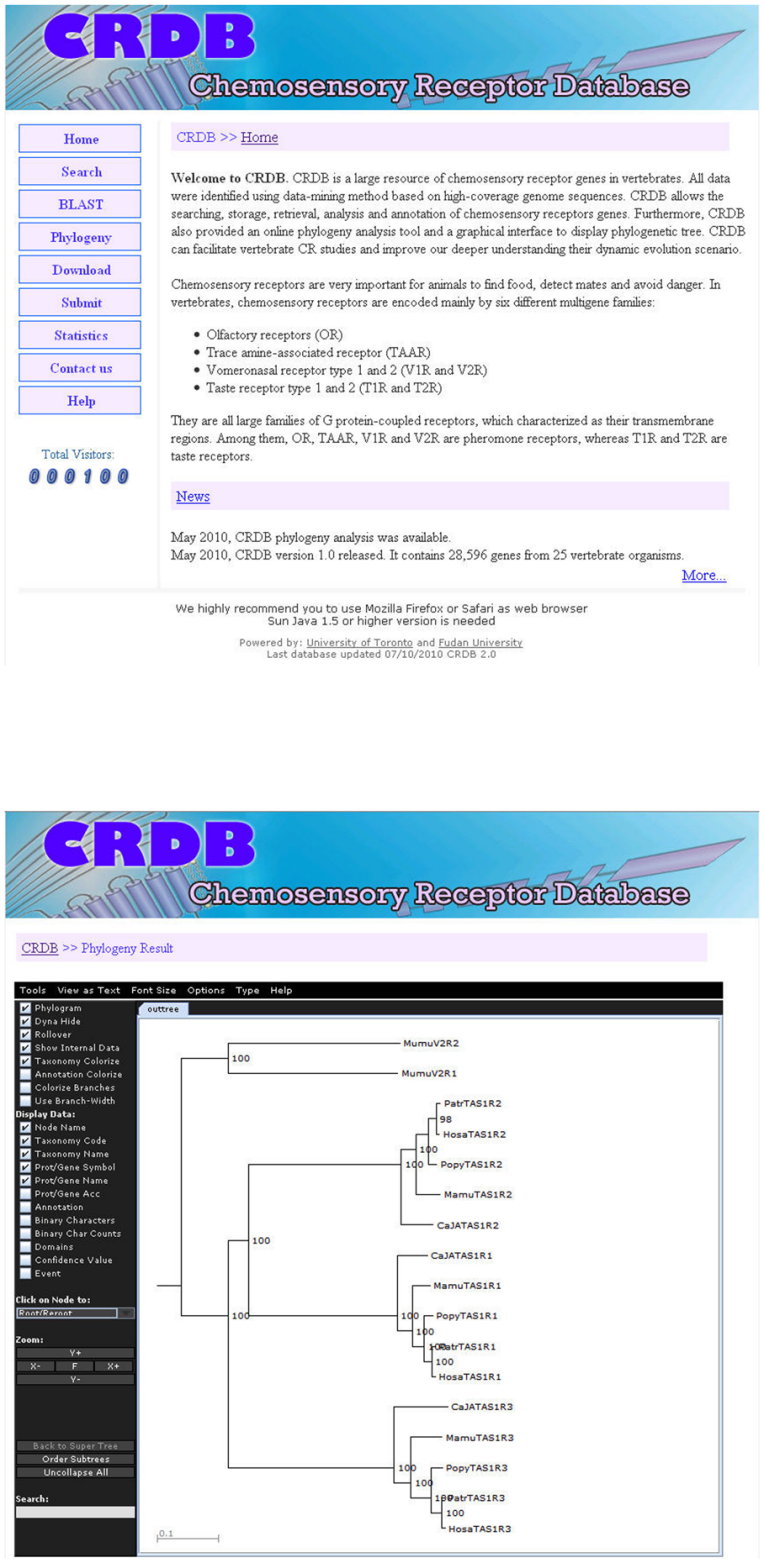
The web interface of CRDB. A) Snapshot of the CRDB home page. B) Result of phylogenetic tree of primate T1R genes.

To our knowledge, the CR genes we identified are the largest curated dataset identified from high-coverage genome sequences. To facilitate CR gene annotation and evolutionary analysis, we subsequently constructed a database (CRDB, http://zldev.ccbr.utoronto.ca/CRDB/). The current version of CRDB (**Figure 2A**) provides a user friendly search engine, which allows to search the content of CR genes using ‘Basic search’ or ‘Advanced search’. In ‘Basic search’, search options for CR genes include by gene name, common name or aliases. Many species have lineage specific CR genes generated from gene duplication. Therefore, these CR genes tend to be located into clusters on specific regions of the same chromosome. For example, T2R genes in the mouse and rat are mainly located on chromosomes 6 and 4 [14], respectively. CRDB allows users to search CR genes at a specific chromosome region of interests in ‘Advanced search’. The system provides details of a specific CR gene including gene annotation, sequence information and cross-reference links. Moreover, we predicted the transmembrane topology of these CR proteins using TMHMM [36], and the transmembrane helix information is provided. Furthermore, to facilitate researchers to submit their identified CR genes to our database, we have implemented a tool for researchers to directly submit their data.

To better understand the phylogenetic relationship among CR genes, CRDB provides an online phylogeny tool. Here, we only confined the phylogenetic analysis to intact CR genes, because most pseudogenes contained deletions and were much shorter than intact genes. Considering the accuracy and efficacy, NJ method was implemented. We have adapted phyloXML [37] for the online phylogenetic tree visualization. **Figure 2B** illustrates an example of phylogenetic tree of T1R genes in five primate species. Mouse V2R sequences were used as the outgroup of the phylogenetic tree. The result showed that primates T1Rs form three monophyletic clades (T1R1, T1R2 and T1R3 clades) with high bootstrap values.

### Conclusion

In this study, we identified nearly the entire CR genes based on 25 vertebrate high-coverage genome sequences. Our results strongly suggest the numbers of CR genes are diverse in vertebrates. In addition, it can also provide us a preliminary scenario for the extensive expansions and contractions of CR genes in vertebrate evolution. The extensively varying numbers of CR genes might be responsible for the species-specific ability of odor and taste perceptions [2]. Our work can be very useful for several reasons. First, we comprehensively identified CR genes in 25 vertebrates, and illustrated a dynamic fluctuating numbers of CR genes. Second, these identified CR genes provide a large resource for further analysis. At last, our CR gene database provides a platform for CR genes, and can facilitate CR gene evolutionary researches in vertebrates.

## Materials and Methods

### Genome sequences

The high coverage genome sequences of 25 vertebrate were downloaded from UCSC genome browser (http://genome.ucsc.edu/), which include human (Homo sapiens, hg19), chimpanzee (Pan troglodytes, panTro2), orangutan (Pongo pygmaeus abelii, ponAbe2), rhesus (Macaca mulatta, rheMac2), marmoset (Callithrix jacchus, calJac1), mouse (Mus musculus, mm9), rat (Rattus norvegicus, rn4), guinea Pig (Cavia porcellus, cavPor3), dog (Canis lupus familiaris, canFam2), panda (Ailuropoda melanoleuca, ailMel1), horse (Equus caballus, equCab2), cow (Bos Taurus, bosTau4), elephant (Loxodonta africana, loxAfr3), opossum (Monodelphis domestica, monDom5), platypus (Ornithorhynchus anatinus, ornAna1), chicken (Gallus gallus, galGal3), zebra finch (Taeniopygia guttata, taeGut1), lizard (Anolis carolinensis, anoCar1), x. tropicalis (Xenopus tropicalis, xenTro2), zebrafish (Danio rerio, danRer6), tetraodon (Tetraodon nigroviridis, tetNig2), fugu (Takifugu rubripes, fr2), stickleback (Gasterosteus aculeatus, gasAcu1), medaka (Oryzias latipes, oryLat2), sea lamprey (Petromyzon marinus, petMar1).

### Identification of CR genes

Semi-automatic data-mining methods were used to identify CR genes from these whole-genome sequences. At first, we collected previously published CR proteins as query sequences [14], [20], [21], [23], [25], [26], [27]. Then, we conducted TBLASTN searches using the E-value 1e-10 against each genome sequence for different types of CR proteins, respectively. Many TBLASTN query hits might located at the same genomic regions, and we extracted non-overlapping sequences showing the lowest E-value among the hits to a given region. Each of the blast hit sequence was extended in both 3′ and 5′ directions along the genome sequences. Among CR genes, the OR, TAAR, V1R and T2R genes lack introns in their coding regions. The coding sequences with proper ATG and the stop codon were directly extracted, and they were regarded as intact CR genes; Sequences that contain interrupting stop codons or frameshifts were regarded as pseudogenes; Remaining sequences containing either initiation codons or stop codons were not considered. T1R and V2R genes contain many introns, and we conducted a cDNA-to-genomic alignment using Spidey to identify the exon/intron genomic structure [38]. At last, all identified sequences were confirmed by BLASTP searches against the NCBI database to ensure that genuine CR sequences were obtained.

### Database construction

We developed a web interface (CRDB) to facilitate online search and phylogeny study of CR genes. CRDB was designed using Java language that provides access to a local MySQL database. To perform the online phylogenetic analysis of CR genes, MAFFT 6.717 [39] was used to generate multiple sequence alignment. The Neighbor-Joining (NJ) method, implemented by QuickTree [40], was used to generate phylogenetic result due to its good balance between accuracy and efficiency. Then, phyloXML were implemented for online phylogenetic tree visualization [37].
